# Expression of Multiple Transgenes from a Single Construct Using Viral 2A Peptides in *Drosophila*

**DOI:** 10.1371/journal.pone.0100637

**Published:** 2014-06-19

**Authors:** Richard W. Daniels, Adam J. Rossano, Gregory T. Macleod, Barry Ganetzky

**Affiliations:** 1 Laboratory of Genetics, University of Wisconsin-Madison, Madison, Wisconsin, United States of America; 2 Department of Physiology, University of Texas Health Science Center at San Antonio, San Antonio, Texas, United States of America; Columbia University, United States of America

## Abstract

Expression of multiple reporter or effector transgenes in the same cell from a single construct is increasingly necessary in various experimental paradigms. The discovery of short, virus-derived peptide sequences that mediate a ribosome-skipping event enables generation of multiple separate peptide products from one mRNA. Here we describe methods and vectors to facilitate easy production of polycistronic-like sequences utilizing these 2A peptides tailored for expression in Drosophila both *in vitro* and *in vivo*. We tested the separation efficiency of different viral 2A peptides in cultured Drosophila cells and *in vivo* and found that the 2A peptides from porcine teschovirus-1 (P2A) and *Thosea asigna* virus (T2A) worked best. To demonstrate the utility of this approach, we used the P2A peptide to co-express the red fluorescent protein tdTomato and the genetically-encoded calcium indicator GCaMP5G in larval motorneurons. This technique enabled ratiometric calcium imaging with motion correction allowing us to record synaptic activity at the neuromuscular junction in an intact larval preparation through the cuticle. The tools presented here should greatly facilitate the generation of 2A peptide-mediated expression of multiple transgenes in Drosophila.

## Introduction

Co-regulated expression of genes is important in many biological contexts and also has great utility when introducing exogenous genes into model organisms. An obvious application is to co-express a reporter gene with an effector to identify or “label” the cells manipulated by the effector gene. Co-expression of several proteins may also be required to produce a desired biological effect. To achieve expression of multiple transgenes in cultured cells, separate plasmids, each with their own promoter, are co-transfected often in combination with a reporter plasmid at a lower concentration. Since the majority of cells that take up DNA will take up both plasmids, reporter-expressing cells are also likely to express the co-transfected genes. While co-transfection with multiple plasmids is possible for *in vitro* cell transfection and even *in vivo* through electroporation, it is far from ideal for most *in vivo* applications as it can still result in genetically manipulated cells that are not marked by a reporter.

Internal ribosome entry site (IRES) sequences have been used as an alternate method to separate two coding sequences under the control of a single promoter. In theory both proteins could be expressed in the same cells and at the same level, but in reality the level of the second protein is often significantly reduced ([1]). In addition, the length of the IRES sequence can be prohibitive in experiments using viruses with small packaging capacity.

The discovery of viral 2A peptides and their use in generating multiple separate proteins from a single mRNA circumvents many of these previous difficulties. The first such peptide characterized is the 2A peptide from foot-and-mouth disease virus (FMDV; [2]), which separates the 2A and 2B protein products. Originally thought to encode a protease cleavage site, the “cleavage” of proteins separated by the 2A sequence surprisingly occurs co-translationally, in an unconventional process where a peptide bond often fails to form between the glycine and terminal proline tRNA in the 2A peptide motif (D(V/I)EXNPG↓P, where ↓ represents the skipped peptide bond). Despite this failure, translation can proceed and two distinct proteins are produced with equimolar stoichiometry. However, this bond skipping does not always occur; if a bond forms, a stable fusion protein is generated that will not subsequently cleave ([3]). A short stretch of the 2A peptide sequence coding for approximately 20 amino acids is sufficient to cause the bond-skipping effect, and similar sequences have been found in several other viruses; the most commonly used versions are from equine rhinitis A virus (ERAV; E2A), FMDV (F2A), porcine teschovirus-1 (P2A), and *Thosea asigna* virus (TaV; T2A).

Previous work demonstrated that the various 2A peptides have different efficiencies of peptide bond skipping and that these efficiencies vary depending on the experimental context. For example, the 2A peptide from FMDV was reported to exhibit ∼90% skipping efficiency ([4]), compared with reports of nearly 100% ([5]), and most recently only 40% ([6]). This variability is almost certainly due in part to differences in experimental conditions, including use of different model organism or cell line. With this in mind, we tested which of the four most widely used 2A peptides work best in Drosophila, both in the S2 cell line and *in vivo* for the efficient expression of multiple polypeptides from a polycistronic mRNA transcribed from a single promoter. As a demonstration of the utility of this approach, we use the P2A peptide to co-express the red fluorescent protein tdTomato and the genetically-encoded calcium indicator GCaMP5G to enable ratiometric calcium imaging at the larval neuromuscular junction (NMJ). The tools presented here should greatly facilitate the generation of 2A peptide-mediated expression of multiple transgenes in Drosophila.

## Materials and Methods

### DNA Cloning

DNA sequences coding for codon-optimized 2A peptides were generated from the oligonucleotides in Table 1, adding 3 amino acids to the N-terminus and 4 amino acids to the C-terminus of the peptide ([7]) (BamHI and BglII sites underlined). 2A peptide sequence variants used are shown in Table 2. 2A sequences were generated by annealing oligos, cutting with BamHI and BglII and cloning into the BglII site of pC5-Kan ([8]) to produce pC5-Kan 2A. This vector contains 18 commonly used restriction enzyme sites N-terminal to the 2A sequence and 9 additional C-terminal sites that facilitate cloning genes of interest on both sides of the 2A sequence, as well as rare 8-cutter sites for shuttling the gene-2A-gene cassette into the pUAS-C5 ([8]) expression vector.

**Table 1:** Oligonucleotides used in this paper (added restriction enzyme sites underlined).

Name	Sequence
F-E2A	aaaggatccGGCCAGTGCACCAACTACGCCCTGCTGAAGCTGGCCGGCGACGTGG
R-E2A	tttagatctGGGGGCGGGGCCGGGGTTGGACTCCACGTCGCCGGCCAGCTTCAGC
F-F2A	aaaggatccGGCGTGAAGCAGACCCTGAACTTCGACCTGCTGAAGCTGGCCGGCG
R-F2A	tttagatctGGGGGCGGGGCCGGGGTTGGACTCCACGTCGCCGGCCAGCTTCAGCAGGTCG
F-P2A	aaaggatccGGCGCCACCAACTTCTCCCTGCTGAAGCAGGCCGGCGACGTGGAGG
R-P2A	tttagatctGGGGGCGGGGCCGGGGTTCTCCTCCACGTCGCCGGCCTGC
F-T2A	aaaggatccGGCGAGGGCCGCGGCTCCCTGCTGACCTGCGGCGACGTGGAGG
R-T2A	tttagatctGGGGGCGGGGCCGGGGTTCTCCTCCACGTCGCCGCAGGTCAGC
F-attB NdeI	GGAACCCATATGGTCGACGATGTAGG
R-attB NdeI	GGCTAGACATATGCTCGACATGCCCGCCG
F-myrGFP BclI	aaatgatcaATCAAAATGGGCAACAAATGCTG
R-GFPo StuI	aaaaggcctCTTGTAGAGCTCATCCATGCCGTG
F-dsRed BamHI	Aaggatccatggcctcctccgagg
R-dsRed XbaI	Gatctagagtcgcggccggc
F-tdTomato BamHI	aaaggatccATGGTGAGCAAGGGCGAGG
R-tdTomato HpaI	aaagttaacCTTGTACAGCTCGTCCATGCCG
F-GCaMP5G BamHI	aaaggatccATGGGTTCTCATCATCATCATCATCATGG
R-GCaMP5G XbaI	aaatctagaTCACTTCGCTGTCATCATTTGTACAAACTC
F-act5c EcoRI	caggaaacagctatgaccatgattacgaattc
R-act5c NotI	aaagcggccgcggtctctggattagacgactgctgg

**Table 2:** 2A peptide sequences.

E2A	QCTNYALLKLAGDVESNPGP
F2A	VKQTLNFDLLKLAGDVESNPGP
P2A	ATNFSLLKQAGDVEENPGP
T2A	EGRGSLLTCGDVEENPGP

*MyrGFP* was created by PCR using F-myrGFP BclI and R-GFPo StuI and pJFRC19 template ([9]) which contains a fly codon-optimized myristylation sequence derived from the first 85 amino acids of the Drosophila Src homolog (Src64B) followed by a codon-optimized EGFP (F64L, S65T). This PCR product was cut with BclI and StuI and ligated with pC5-Kan 2A cut with BamHI/StuI.

*DsRed-nls* is also called *RedStinger* ([10]), which is a DsRed variant containing a nuclear localization signal from the Drosophila *transformer* gene. It was amplified by PCR with F-dsRed BamHI and R-dsRed XbaI, and cloned into pCR8GW TOPO, then excised with BamHI/XbaI and cloned into pC5-Kan *myrGFP-2A* cut with BglII/AvrII to make pC5-Kan *myrGFP-2A–RedStinger*.

pPac-C5 was generated by modifying the MCS from pPac-PL (C.S. Thummel, unpublished). A BamHI/AvrII fragment from pC5-Kan was inserted into BamHI/XbaI sites of pPacPL to make pPac-C5 with a MCS including the following unique restriction sites: BamHI, XbaI, XhoI, StuI, AgeI, MluI, NheI, KpnI, NotI.

A SpeI/AscI fragment from pC5-Kan *myrGFP-2A–RedStinger* was then cloned into XbaI/MluI sites of pPac-C5 to generate pPac-C5 *myrGFP-2A–RedStinger*.

To make pPac-C5 myrGFP-dsRed-nls (no 2A peptide), myrGFP was subcloned from pC5-Kan myrGFP P2A into pBS-C5 ([8]) using EcoRI/StuI. Then dsRed-nls PCR cut with BamHI/XbaI (as described above) was added into BglII/NheI sites of pBS-C5 myrGFP. myrGFP-dsRed-nls was then cut with SpeI/AscI and ligated with pPac-C5 cut with XbaI/MluI to generate pPac-C5 myrGFP dsRed-nls (no 2A peptide control).

pUAS-C5 attB was made by inserting a 285 bp attB site created from PCR with F-attB NdeI and R-attB NdeI using pattB as template ([11]), then inserted into NdeI site of pUAS-C5 ([8]).

A SpeI/AscI fragment from pC5-Kan *myrGFP-2A–RedStinger* was subcloned into pUAS-C5 attB cut with SpeI/AscI and injected into a fly harboring the p{CaryIP}Su(hw)attP1 site at 87B13 on the third chromosome using standard techniques to generate UAS-*myrGFP-2A–RedStinger* flies.

To make pUAS-C5 attB *tdTomato-P2A–GCamP5G*, *tdTomato* was made by PCR with F-tdTomato BamHI and R-tdTomato HpaI using pRSET-B *tdTomato* template (R. Tsien; [12]), cut with BamHI/HpaI and inserted into the BamHI/StuI sites of pC5-Kan *P2A*. *GCamP5G* was amplified from Addgene plasmid #31788 with F-GCaMP5G BamHI and R-GCaMP5G XbaI, then cut with BamHI/XbaI and inserted into BglII/AvrII sites of pC5-Kan *tdTomato P2A*. The *tdTomato-P2A–GCaMP5G* fragment was then subcloned into pUAS-C5 attB with PacI/AscI and injected into p{CaryIP}Su(hw)attP1 site at 87B13 on the third chromosome.

Act5c-LexAGADfl flies were generated by amplifying the act5c promoter using F-act5c and R-act5c primers, cloning into pCR8-GW-TOPO (Invitrogen), and performing an L/R reaction with pBPnlsLexAGADflUw ([9]). The resulting construct was integrated into the p{CaryP}attP40 site at 25C6 on the second chromosome.

pC5-Kan P2A has been deposited with Addgene (plasmid #70838) and GenBank (KJ470630). Other plasmids, sequences, and flies are available upon request.

### Fly Stocks

The following genotypes were used: *act5c*-Gal4 (stock #3954, deposited by Dr. Yashushi Hiromi, unpublished), tubulin-Gal4 (stock #5138, deposited by Dr. Liqun Luo ([13]) and *n-syb*-Gal4 (stock #51635, generated by Dr. Julie Simpson, unpublished) (from Bloomington stock center). UAS-*myrGFP-2A–RedStinger* and UAS-*tdTomato-P2A–GCaMP5G* flies were generated as described above. Act5c-LexA (this paper) and LexAop:myrGFP (stock #32212) were used as negative controls for the western blot from adult flies.

### S2 Cell Culture

S2 cells (selected for adhesion; gift of Dr. Cedric Wesley, University of Wisconsin-Madison) were grown in DMEM (Invitrogen) supplemented with 10% FBS and 1% penicillin-streptomycin and transfected using Effectene (QIAgen) according to manufacturer’s protocol.

### Western Blotting

S2 cells were harvested, washed with PBS and resuspended in 2x SDS sample buffer, then boiled and centrifuged. Supernatant was then loaded on a 14% SDS-PAGE gel. 15 adult flies were anesthetized, transferred to tubes and homogenized in 2x SDS sample buffer, boiled and centrifuged. Approximately 1 fly was loaded per lane.

Protein was transferred to a PVDF membrane, blocked with Odyssey blocking buffer (Licor) and probed with rabbit anti-GFP (A6455, Invitrogen) at 1∶1000, followed by donkey anti-rabbit 800 IRDye (Licor) at 1∶10,000. Quantitative imaging was performed using an Odyssey infrared imaging system (Licor). Integrated intensity measurements were performed with Odyssey software. Separation efficiency was calculated as the lower band intensity divided by the sum of lower and upper band intensities after subtracting the background.

### Immunohistochemistry and Imaging

S2 cells were harvested 48 hours post-transfection and resuspended in fresh media and then plated on a poly-lysine coated coverslip for 10 minutes. The solution was removed and replaced with PBS and then 4% parafomaldehyde in 0.1 M phosphate buffer, pH 7.20 for 5 minutes. Fixative was removed and the coverslip washed with PBS before placing on a droplet of Vectashield (Vector Laboratories) on a slide and sealed with nail polish.

3^rd^ instar larval salivary glands were fixed 20 minutes in 4% PFA freshly diluted from 16% stock (EM Sciences) in 0.1 M phosphate buffer pH 7.20 with 0.2% Triton-X 100, then washed with PBS containing 0.1% Triton-X 100 and To-Pro3 (Invitrogen) before equilibrating in 70% glycerol and mounting in Vectashield.

Samples were imaged on a Zeiss LSM 510 confocal microscope with an Imager.M1 module using 20X/0.8 NA Plan-APOCHROMAT, 40X/1.3NA Plan-NEOFLUAR or 63X/1.4NA Plan-APOCHROMAT objectives. Fluorescence was excited using an Argon laser at 488 nm, a DPSS 561 laser at 561 nm, and a HeNe laser at 643 nm.

### Image Analysis

The 3D Object Counter plugin in ImageJ software (FIJI package,NIH) was used to determine the size and abundance of bright GFP/RFP-positive aggregates from confocal stacks of larval muscles.

### Live Imaging

Live imaging was performed *ex vivo* in modified BES-buffered hemolymph-like solution 6 (0mM NaHCO_3_^−^, 19mM BES, pH 7.3; [14]) supplemented with 2mM Ca^2+^ and 7mM L-glutamic acid (LGA; Cat. no. G1626; Sigma-Aldrich) to prevent muscle contraction during nerve stimulation.

Wide-field microscopy was performed on an Olympus BX51WI microscope fitted with a 60× (0.9 NA) water-immersion objective. A Lumen Dynamics (Mississauga, Ontario, Canada) X-Cite XLED1 LED excitation unit with GYX (540–600 nm) and BDX (450–495 nm) modules was used to supply excitation light which was then further filtered with a dual-band pass 470/556 nm filter. Images were captured with an Andor Technology (Belfast, Northern Ireland) EMCCD camera (DV887). A Cairn Instruments (Kent, UK) Optosplit II emission beam-splitter was placed before the camera for dual-emission wavelength imaging. Simultaneous imaging of tdTomato (ex./em. 556/640 nm, [12]) and GCaMP5 (ex./em 480/530 nm, [15]) was achieved using a primary 500/530 nm bandpass dichroic mirror in the microscope housing, a secondary 600 nm dichroic mirror mounted in the emission beam-splitter, and 512/25 nm and 660/20 nm emission filters in the beam-splitter. Filters and dichroic mirrors were obtained from Chroma Technology (Bellows Falls, VT, USA) or Semrock (Lake Forest, IL, USA). The imaging system was controlled through a Dell PC running Andor iQ software (version 2.7.1). All imaging was performed at room temperature (∼24°C) on MN termini of axons innervating muscle 13 of abdominal segment 4 with type-Ib ‘big’ boutons (MN13-Ib), with regions of interest drawn around at least three boutons. The image acquisition rate was 5 Hz in all cases.

### Preparation of Specimens

For *ex vivo* imaging, female wandering 3^rd^ instar Drosophila larvae were collected and fillet-dissected in chilled HL6 to expose the longitudinal muscles of the body wall and the segmental nerves that project from the ventral ganglion. The posterior ventral ganglion was cut from the cerebral ganglia to stop endogenous nerve activity ([17]) and a loop of a segmental nerve was drawn into the lumen of a glass micropipette with the ventral ganglion still attached ([14]). Nerves were stimulated as described previously ([18]). For *in* *vivo* imaging, intact larvae were restrained between a glass slide and coverslip.

### Statistical Analysis and Figure Preparation

SigmaPlot (version 12.3; Systat Software) was used for statistical analysis. One-way ANOVA (with a Bonferroni *post hoc* test) was used for comparisons. Differences were considered to be statistically significant at α-values of *P*≤0.05.

## Results

### A Versatile Shuttle Vector for Cloning 2A Peptides

**Figure 1:**
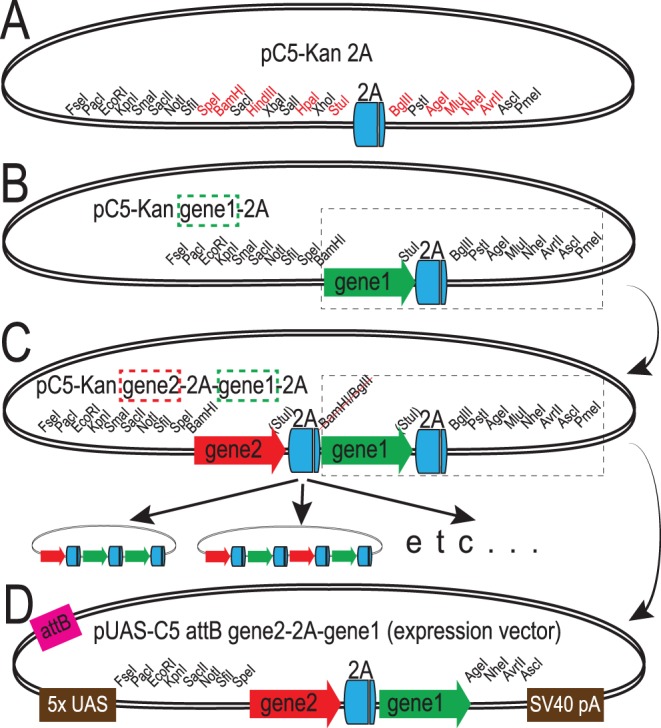
Outline of vector design for cloning 2A peptide-linked transgenes. A. Diagram of pC5-Kan 2A, a vector containing a codon-optimized 2A peptide coding sequence flanked on each side by multiple cloning sites that facilitates cloning. Restriction sites in frame with the 2A sequence are shown in red. B. General procedure for making multimeric 2A-linked transgenes. A fragment cut at the 5′ end with BamHI and at the 3′ end with any of the unique restriction enzymes following the 2A coding sequence (boxed region) can then be inserted 3′ of the 2A sequence in another vector cut with BglII and the second chosen enzyme, creating the vector shown in panel C. This process creates a BamHI/BglII hybrid site that is in frame with the first coding sequence (red arrow), and leaves the BamHI and BglII sites unique so they can be used again if required. The process of adding a gene-2A cassette can be repeated using this method to create the desired construct. Examples are shown at the bottom. D. Diagram of an expression vector demonstrating the final step of the cloning procedure. The pUAS-C5 vector contains the same multiple cloning site as the pC5-Kan shuttle vector and has been modified with an attB site for phiC31-mediated integration into genomic attP sites.

As a first step to compare the properties of different 2A peptide sequences in Drosophila, we generated a vector that facilitates cloning of transgenes separated by the 2A sequence. This set of vectors contains a 2A peptide sequence in the middle of two multiple cloning sites and is based on the C5 series of vectors ([8]). They have a large number of unique restriction enzyme cut sites that make it easy to transfer cloned DNA from small shuttle vectors into injectable expression vectors for *in vivo* transformation of Drosophila. A diagram of this vector and the restriction enzyme sites are shown in Figure 1. As an additional feature, we designed the vector so that once a gene is added 5′ to the 2A sequence, it can be easily removed and added downstream of the 2A sequence in another vector (Figure 1B and 1C). This modular design with gene-2A cassettes can improve cloning efficiency and enable rapid construction of chains of transgenes that can be expressed together from the same promoter. After the desired construct has been generated in the pC5-Kan 2A shuttle vector, it can be easily subcloned into the pUAS-C5 expression vector using flanking rare restriction enzymes (Figure 1D). We produced vectors to test four of the most commonly used 2A peptide variants from equine rhinitis A virus (ERAV; E2A), human foot-and-mouth disease virus (FMDV; F2A), porcine teschovirus-1 (P2A), and *Thosea asigna* virus (TaV; T2A).

### Comparison of 2A Peptides *In vitro*

**Figure 2:**
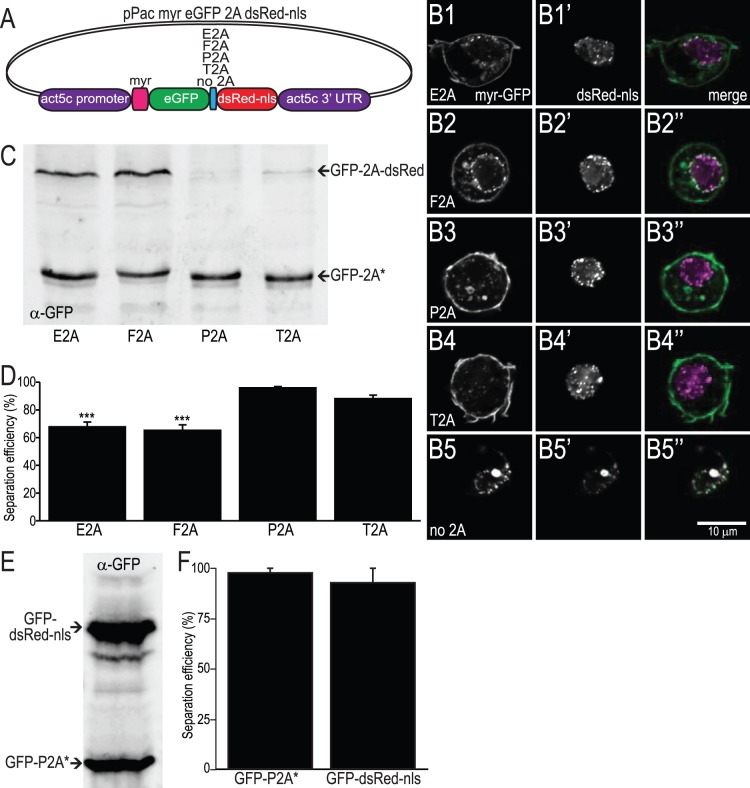
Demonstration of separation of proteins linked by different 2A peptides in cultured cells. A. Diagram of vector used to transform S2 cells to express myristylated GFP and nuclear-localized dsRed. B. Single optical sections from confocal micrographs of fixed S2 cells transfected with vector shown in A. Native GFP and dsRed fluorescence are shown in the leftmost and center panels, respectively. The rightmost panel shows the merged image. While most GFP and dsRed fluorescence is localized correctly, some perinuclear colocalization can be seen. In the absence of a 2A peptide (panel B5), all GFP and dsRed signals colocalize and are enriched in a ring surrounding the nucleus. Similar perinuclear puncta observed in merged image panels (B1’’-B4’’) likely indicate full-length (unseparated) myrGFP-2A-dsRednls protein. C. Example western blot from transfected S2 cells using an anti-GFP antibody demonstrating separation efficiency of different 2A peptides. The top band runs at approximately 75 kDa and is unseparated myrGFP-2A-dsRednls, while the bottom band runs at approximately 37 kDa and is separated myrGFP-2A (GFP-2A*) only. Slight differences in running speed are due to differences in 2A peptide length. D. Histogram showing separation efficiency of 2A peptides tested in S2 cells. Error bars indicate SEM, n = 5. The P2A and T2A peptides yield the most complete separation (one-way ANOVA with Bonferonni post-test; p<0.001 vs. P2A and T2A indicated by asterisks). E. Western blot showing ratiometric expression of proteins before and after 2A peptide. The lower band is GFP-2A and the upper band is GFP-dsRed-nls. F. The intensity of each band was quantified and plotted in the histogram. The average normalized intensity of the lower band is 98+/−2 versus 93+/−7 for the upper band. Error bars indicate SEM, n = 2.

Using the vectors described above, we tested the separation efficiencies with which each of the four most widely used 2A peptides. Previous studies have used a number of different cell-free and cell-based assays to determine separation efficiency and a range of values have been reported for each peptide ([4], [5], [6], [19]). Our strategy was to express a membrane-targeted green fluorescent protein (mGFP) and a red fluorescent protein with a nuclear localization signal (dsRed-nls) separated by the 2A peptide (Figure 2A; see methods for details). Using this approach, we could image 2A-dependent polypeptide separation in fixed cells and also quantify the level of separation using SDS-PAGE followed by western blotting.

We transfected the Drosophila S2 cell line with each of the four versions of mGFP-2A-dsRed-nls, as well as one without any 2A peptide sequence, driven by the ubiquitous *actin5c* promoter. Figure 2B shows sample confocal micrographs of GFP and RFP fluorescence in fixed S2 cells after transfection. Since any fusion protein resulting from failure of the 2A peptide bond-skipping process will contain both a membrane localization signal (N-terminal myristyl group) and a nuclear localization signal, we expected to detect these full-length polypeptides in nuclear or perinuclear domains. In the absence of a 2A peptide, GFP and RFP fluorescence is concentrated in perinuclear puncta (figure 2 B5). We observe similar perinuclear puncta more frequently in cells transfected with E2A- and F2A-containing vectors than with the P2A and T2A vectors, indicating that E2A and F2A have lower separation efficiency in Drosophila S2 cells. More quantitatively, we measured separation efficiency by western blots using an antibody against GFP. As noted previously ([2]), we detect the presence of slower-migrating bands of the size expected for a full-length mGFP-2A-dsRed-nls protein product, and the relative abundance of this upper band varies depending on the particular 2A peptide sequence; the P2A and T2A peptides yield the best separation efficiency (Figure 2C and D). E2A had an efficiency of 68+/−3%, compared with 65+/−4% for F2A, 96+/−1% for P2A and 88+/−3% for T2A (average +/− SEM, n = 5, p<0.001 by one-way ANOVA between E2A and F2A compared with either P2A or T2A; P2A and T2A were not significantly different).

The unconventional way in which the second polypeptide is generated using 2A peptides results in an N-terminal proline which could limit the protein’s perdurance. Additionally, the ribosome could disassociate when encountering the conserved NGPG motif, leading to reduced translation of the second protein. To test whether the protein following the 2A sequence is expressed ratiometrically, we transfected S2 cells with a construct encoding GFP-P2A–GFP-dsRednls. In this way we can directly compare the GFP-2A* (separated) band with a higher GFP band whose apparent molecular weight has been shifted by fusion with dsRed. A western blot probed with anti-GFP shows both bands are present with similar intensities (figure 2E). This is quantified in figure 2F, demonstrating that the proteins are produced at very similar levels (n = 2).

### Comparison of 2A Peptides *In vivo*

**Figure 3:**
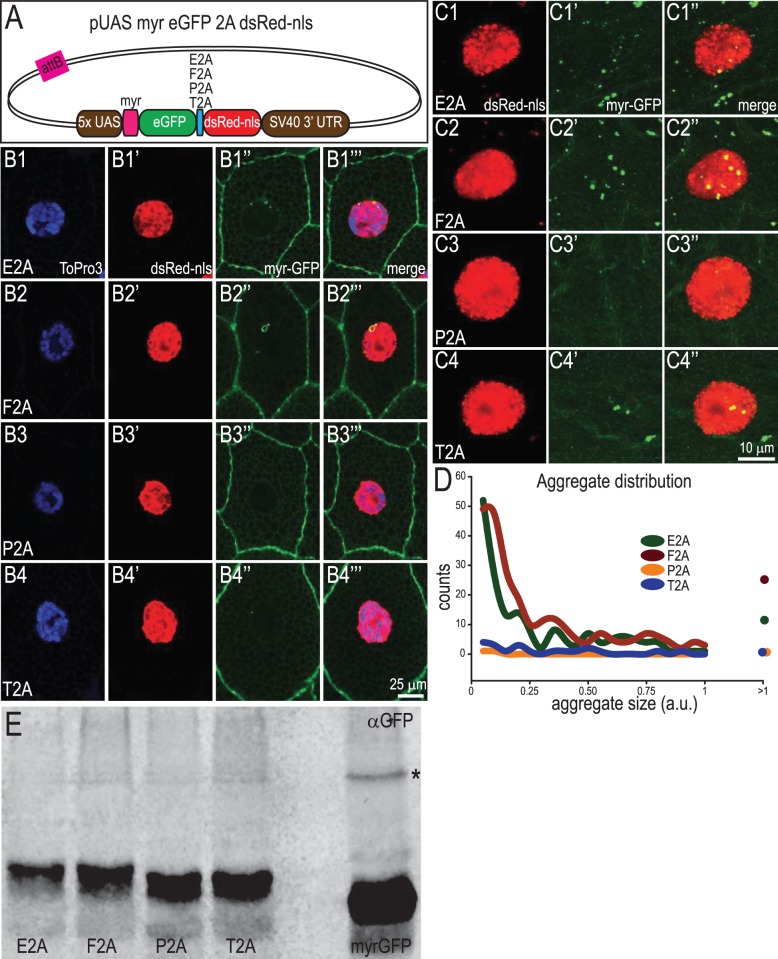
Testing the separation efficiency of 2A peptides *in vivo*. A. Diagram of the DNA construct injected into flies placing expression of myrGFP-2A-dsRed-nls under the control of Gal4. B. Single confocal sections showing separation of myrGFP and dsRed-nls in salivary gland cells from third instar larvae in which UAS-myrGFP-2A-dsRednls is driven by *act5c*-*Gal4*. The first panel for each peptide version shows DNA (stained with ToPro3), the second panel shows dsRed-nls fluorescence, the third shows GFP fluorescence, and the last panel a merged image of all three channels. C. Volume maximum intensity projection of confocal micrographs from larval muscle cells expressing myrGFP-2A-dsRed-nls driven by *how^24B–^Gal4*. DsRed and GFP fluorescence are shown in the first two panels, respectively, and the third panel shows the merged image. Unseparated protein products appears as aggregates. D. Analysis of dsRed/GFP aggregates in muscle cells. More and larger aggregates are detected in cells expressing E2A- and F2A-linked transgenes, N = 2 cells per genotype. E. Western blot from adult fly tissue expressing myrGFP-2A-dsRed-nls driven by *tubulin-Gal4* or LexAop-myrGFP driven by *act5c-LexA* (last lane) as a negative control. The upper band representing unseparated protein product cannot be seen at this exposure (asterisk is next to non-specific band also seen when myrGFP is expressed alone), despite prevalence of lower band representing separated GFP-2A* protein.

To test how the 2A peptides performed *in vivo*, we generated transgenic fly lines where the mGFP-2A-dsRed-nls construct was placed under the control of the yeast upstream activation sequence (*UAS*) so that it could be expressed under the control of tissue-specific *Gal4* lines (Figure 3A). To control for position effects on transgene expression level, we integrated all four constructs into the same genomic locus using the phage phiC31 system ([20], [9]). Separation of GFP and RFP was best demonstrated in large cells with good separation between membrane and nuclear compartments, such as larval salivary gland and somatic muscles. When we express these transgenes in the larval salivary gland, we can easily see differences among the different 2A peptides in accumulation of perinuclear GFP/RFP-positive puncta (Figure 3B). Similar to the results with S2 cells, expression utilizing E2A and F2A peptides in salivary glands results in the most prevalent production of perinuclear GFP/RFP-positive puncta, which indicate failure of the peptide-bond skipping event. In contrast, we observe few of these puncta in cells expressing the constructs containing the P2A and T2A peptides, indicating a high efficiency of polypeptide separation *in vivo* (figure 3B). When DsRed-nls was expressed alone in the salivary gland cells we do not observe extra-nuclear spots of red fluorescence, suggesting that these accumulations result from mis-targeting due to the myristylation sequence of mGFP (data not shown). Differences in separation efficiency are recapitulated with expression in other cell types including larval body wall muscles (figure 3C) and larval multi-dendritic sensory neurons (data not shown). The analysis of GFP/RFP-positive aggregate number from muscle cells shown in figure 3D demonstrates that increased numbers of larger aggregates are found in cells expressing E2A- and F2A-containing transgenes.

We also analyzed separation efficiency of 2A-linked fluorescent proteins by western blot analysis of whole fly extracts. Surprisingly, the band representing full length myrGFP-2A-dsRed-nls was present at a much lower level than in S2 cell extracts (figure 3E). This observation suggests that some type of clearance mechanism is operating to eliminate the myrGFP-2A-dsRed-nls fusion protein, perhaps because of the competing localization signals.

### Using P2A to Facilitate Ratiometric Calcium Imaging

The invention of optogenetics and the evolution of fluorescent reporters and sensors in recent years have provided strong impetus to develop efficient new ways of applying these tools to biological problems. In many instances, significant experimental advantages are gained by expression of multiple reporters and sensors in the same cell, such as the paired expression of channelrhodopsin and halorhodopsin for dual color optical control of electrical activity ([21]). Another example is tandem expression of a fluorescent indicator along with a control fluorescent protein, enabling measurement of baseline differences and comparison between cells and experiments.

Because of the very high polypeptide separation efficiency of the P2A peptide, we used it to develop a tool for *in vivo* calcium imaging. The genetically encoded calcium indicator GCaMP has undergone a series of refinements since its invention, with the most recent versions offering increased signal-to-noise ratios (SNR) by 2–3 fold ([15] [16]). Unfortunately, this increased SNR has been primarily achieved by significantly lowered basal fluorescence, which can make it difficult to focus on a region of interest before and even during calcium imaging, especially if there is any movement of the preparation during recording. We sought to circumvent these problems by co-expressing a bright and stable red fluorescent protein along with one of the newer GCaMP indicators. We therefore made a construct that expresses the red fluorescent protein tdTomato followed by the P2A peptide and then GCaMP5G, all under the control of the *Gal4/UAS* system.

**Figure 4:**
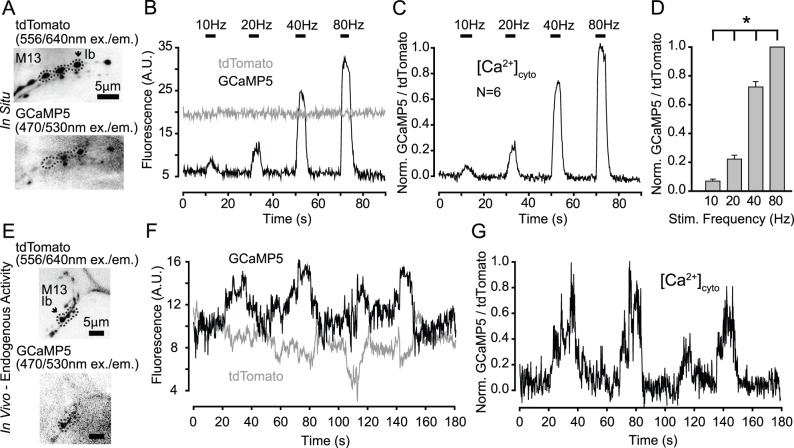
Application of the P2A peptide system for ratiometric calcium imaging at the larval NMJ. A. Dual-channel imaging of tdTomato (top) and GCaMP5G (bottom) at the neuromuscular junctions on muscle 13 of a dissected third instar larva expressing UAS-tdTomato-P2A–GCaMP5G driven by *n-syb-Gal4*. B. Fluorescence records from MN13-Ib synaptic boutons responding to electrical stimulation of the segmental nerve at the indicated frequencies. C. GCaMP5G fluorescence data from B normalized to the simultaneously collected tdTomato signal. D. Histogram showing peak normalized GCaMP5G fluorescence intensity as a function of stimulation frequency (n = 6 larvae, mean +/− SEM). E. Dual-channel imaging of tdTomato (top) and GCaMP5G (bottom) at the neuromuscular junctions on muscle 13 taken through the cuticle of an intact larva. F. Fluorescence records from synaptic boutons responding to endogenous bursts of action potential firing. Note the large fluctuations in tdTomato signal that are indicative of muscle contraction and associated movement artifacts. G. GCaMP5G fluorescence from panel F normalized to tdTomato signal, which reveals a more biologically-relevant signal.

We expressed *UAS-tdTomato-P2A–GCaMP5G* in motoneurons and recorded red and green fluorescence intensities with muscle contraction blocked in a dissected larval preparation. We were easily able to detect calcium flux into the nerve terminals upon electrical stimulation of the axons and the GCaMP fluorescence signal increased with increasing stimulation frequency (Figure 4A and 4B). Because movement of the preparation was blocked, the tdTomato signal shows minimal fluctuation over the duration of the experiment, so correction of the GCaMP signal by normalizing to tdTomato has little effect (Figure 4C). We could also easily quantify peak GCaMP fluorescence intensity at different stimulation frequencies using regions of interest demarcated by tdTomato fluorescence (Figure 4D).

To test whether a strong red signal would allow us to measure changes in the cytoplamsic calcium concentration in a preparation where contraction of body wall muscles was not blocked and movement occurred, we recorded fluorescence intensity from motoneuron terminals through the cuticle of an intact larva (Figure 4E and 4F). Raw GCaMP fluorescence signals recorded in this experimental setup are difficult to interpret alone, but when they are normalized to the tdTomato signal, we can distinguish real calcium-dependent signals from movement-derived artifacts (Figure 4G). Thus, by use of the P2A peptide vector, we were able to achieve a significant refinement over existing imaging techniques demonstrating an example of the useful ways in which this technology can be applied.

## Discussion

Expression of multiple polypeptides at similar levels from a single mRNA is made possible by inclusion of a short viral 2A peptide coding sequence between the polypeptide-encoding transgenes. Here we compare four of the most commonly used 2A peptides from different viruses and find that the P2A and T2A peptides exhibit the highest efficiency of polypeptide separation in Drosophila both in cultured cells and *in vivo* enabling separation of co-expressed, differentially targeted fluorescent proteins. We also demonstrate use of the P2A peptide to improve ratiometric calcium imaging with GCaMP5G. Furthermore, the series of vectors we have developed that incorporate 2A peptides permit easy cloning of 2A-linked transgenes for expression in Drosophila.

### Previous Use of 2A Peptides in Drosophila

The use of 2A peptides in Drosophila has been described in four recent reports. Gonzalez et al., 2011([22]) utilized a 2A peptide-containing vector to co-express a fluorescent marker (GFP or mCherry) and an antibiotic resistance gene (*Neo^R^*) to facilitate the establishment of stable S2 cell lines. They used the 2A peptide from the *Thosea asigna* virus, but did not compare different 2A peptides. The vector used in these experiments only permits cloning a single gene for stable expression in S2 cells, whereas our vectors are more general and make it easy to clone multiple genes back-to-back.

Inagaki et al., 2012 ([23]) utilized a 2A peptide that was not described to co-express two parts of an artificial signaling cascade and did not report any data on separation efficiency.

Diao and White, 2012 ([24]) utilized a 2A peptide to effectively co-express Gal4 from the translational start site of a protein of interest. They compared the same four 2A peptides we studied here as well as the 2A peptide from Drosophila C virus (DCV) and concluded that T2A worked the best. However, their assay was only qualitative and based on examining nuclear versus membrane localization of Gal4 in cultured S2 cells (and the subsequent signal amplification by a *UAS*-reporter). Although we are in agreement that T2A is far better than either E2A or F2A, our quantitative evidence indicates that the P2A peptide is equally efficient as T2A at generating distinct proteins in Drosophila. These minor discrepancies may be due to use of slightly different sequences surrounding the 2A peptide or differences inthe particular assays used.

Finally, Masuyama et al., 2012 ([25]) utilized a reporter with two membrane protein-GFP fusions separated by an undisclosed 2A peptide to increase fluorescence intensity.

Only one of these previously published papers describes a useful vector for cloning, and that vector only enables use of T2A to express a single gene upstream of a resistance gene ([22]). Significant improvements incorporated into our vector include a greater selection of cloning sites before and after the 2A sequence, the capability for easily generating multimeric gene-2A repeats, and the ease with which the finished construct can be shuttled into an expression vector (such as pUAS-C5 attB) for *in vivo* transformation. Using a small shuttle vector for the initial cloning is beneficial in many ways. First, more unique sites are present in the shuttle which increases the likelihood that a gene of interest can be inserted in frame with the 2A sequence. Second, the smaller vector is easier to amplify in bacteria and purify with higher yields which is helpful for sequencing inserts. Third, the inserts from the shuttle vector are generic and can be subcloned into expression vectors with different purposes, for example expression in cultured cells or for transformation of genetic model organisms.

Another method for inserting a 2A peptide would be to directly add the sequence during PCR using an oligonucleotide containing the 2A coding sequence and homology to the gene of interest, as was done by Diao and White ([24]). However, making gene1–2A-gene2 constructs such as those we present here would require multiple PCR steps and may be less efficient than stepwise addition into the pC5-Kan 2A vector. This would be especially true for longer chains of transgenes. We expect that the P2A and T2A versions would still work best if added using PCR.

Of the four vectors we present here, the E2A- and F2A-carrying versions may not be as useful in Drosophila, given their poor efficiency at polypeptide separation. However, the use of both P2A and T2A sequences in a longer construct instead of solely P2A may reduce DNA rearrangements in bacterial plasmids. More recent reports on different versions of F2A have found that longer sequences can perform better ([19]), but we only tested the short 22 amino acid version. Despite significant differences in separation efficiency in S2 cell culture and in many *in vivo* tissues examined, we observe far less unseparated protein *in vivo* than we expected. For example, given the data in figure 2D, we would expect ∼35% of E2A-containing protein to be unseparated. Although we find prominent GFP/RFP-positive aggregates in salivary gland and muscle using immunohistochemistry, they are limited, and western blots show very little protein of the expected size for unseparated myrGFP-2A-dsRed-nls. One explanation for this difference could be that membrane-targeted proteins with nuclear localization signals are selectively degraded by protein quality control pathways that are less efficient in S2 cells or become saturated at high expression levels. Another possibility is that separation efficiency could depend on the tissue or developmental stage, although we think this is unlikely given the qualitative similarities between the diverse cell types we examined. While the apparent dearth of unseparated protein on western blots would on the surface seem to indicate that all 2A peptides are efficient *in vivo*, we caution that this could be an artifact of the particular localization signals we used, and not a general finding. In cell types where we can easily detect aggregated GFP/RFP, the relative accumulation recapitulates the separation efficiency measured in cell culture. Further studies will be needed to investigate separation efficiency *in vivo* in a variety of Drosophila cells as well as at different developmental stages to confirm the generality of our results. To demonstrate the usefulness of the 2A peptide methodology, we have applied it to ratiometric Ca^2+^ imaging at the larval Drosophila neuromuscular junction. Use of the P2A peptide greatly simplified co-expression of tdTomato with GCaMP5G compared with the crossing schemes that would otherwise be required to generate a fly capable of expressing both proteins. The 2A peptide technique also enables the co-expressed proteins to be produced at approximately equal levels, which is an important consideration in a number of applications. Additionally, 2A peptides allow production of multiple proteins using the same promoter sequence, which reduces the dilution of transcription factors that could result from the presence of too many binding sites, as can occur with weakly-expressing Gal4 lines driving multiple *UAS*-linked transgenes. Newer versions of GCaMPs have lower fluorescence in the unbound state, which makes expression of another fluorescent marker protein essential for identifying the particular region or cells selected for experimental analysis. As our results demonstrate, by use of a co-expressed marker protein we can also control for movement artifacts during imaging. Thus, we were able to monitor presynaptic activity at larval NMJ in a living, intact, contracting larval preparation, which is highly desirable in a number of experimental situations.

There are a large number of potential applications for controlled co-expression of multiple polypeptides from a single polycistronic mRNA transcribed from a transgenic construct. Increasingly, new techniques in Drosophila and other genetic model organisms rely on genetic lines harboring multiple transgenes which can be complicated to obtain using standard recombination and crossing schemes. The 2A peptide technology has the potential to reduce this complexity and simplify stock construction. The new vectors we present here greatly facilitate synthesis of 2A peptide-mediated polycistronic constructs enabling convenient and broad application of this technology.
